# Cinnabar-induced hormesis in *Trichoderma longibrachiatum* MD33: multi-omics elucidation of a fungal-specific dendrobine biosynthesis pathway

**DOI:** 10.3389/fmicb.2025.1657982

**Published:** 2025-11-07

**Authors:** Archana Jain, Surendra Sarsaiya, Qihai Gong

**Affiliations:** 1 Key Laboratory of Basic Pharmacology and Joint International Research Laboratory of Ethnomedicine of Ministry of Education, Zunyi Medical University, Zunyi, China; 2 Bioresource Institute for Healthy Utilization, Zunyi Medical University, Zunyi, China

**Keywords:** hormesis, metabolic engineering, secondary metabolism, transcriptomics and metabolomics, stress-induced biosynthesis

## Abstract

**Introduction:**

The endangered orchid *Dendrobium nobile* is the primary source of dendrobine, a neuroprotective sesquiterpene alkaloid, but unsustainable harvesting necessitates alternative production platforms, such as the endophytic fungus *Trichoderma longibrachiatum* MD33. However, the fungal dendrobine pathway and its regulatory mechanisms remain uncharacterized, limiting its biotechnological exploitation.

**Methods:**

This study investigated cinnabar (HgS)-induced hormesis to elucidate the stress-mediated metabolic reprogramming of dendrobine biosynthesis through integrated transcriptomic and metabolomic profiling.

**Results:**

Subtoxic HgS concentrations (1.0–4.0 μg/L) triggered ROS signaling, upregulating the mevalonate pathway, terpenoid synthases (TPS1/2), and cytochrome P450 monooxygenases (*CYP450s*), thereby enhancing dendrobine yields by 24% at 4.0 μg/L. In contrast, cytotoxic doses (6.0 μg/L) suppressed growth (73.9% inhibition) and dendrobine synthesis (73.2% reduction), correlating with metabolic collapse via disrupted CoA biosynthesis and antioxidant depletion. Multi-omics integration revealed biphasic regulation: low HgS activated stress-responsive transcription factors (bZIP, Zn-Cys6) and MAP kinase cascades, redirecting resources toward dendrobine production, whereas high HgS induced apoptotic markers and oxidative damage.

**Conclusion:**

These findings establish 4.0 μg/L a hormetic threshold for maximizing dendrobine yields and delineating the genetic and enzymatic architecture of its fungal biosynthesis. This work provides a roadmap for the metabolic engineering of *T. longibrachiatum* MD33, emphasizing ROS-mediated pathway optimization for sustainable alkaloid production. Future studies should leverage CRISPR-based editing of identified regulatory nodes (e.g., *HMGR* and *FPPS*) to enhance stress resilience and dendrobine titers in industrial strains.

## 1 Introduction

Dendrobine, a pharmacologically active sesquiterpene alkaloid primarily isolated from the endangered orchid *Dendrobium nobile*, has long been valued in traditional medicine for its neuroprotective, antipyretic, and analgesic properties (Bhardwaj et al., 2024; Sarsaiya et al., 2025; Shi et al., 2025). However, the overharvesting of wild *D. nobile* populations and the slow growth rate of cultivated plants have created an urgent need for sustainable alternatives to meet pharmaceutical demand (Cheng et al., 2019). The discovery of dendrobine-producing endophytic fungi, such as *Trichoderma longibrachiatum* MD33, offers a promising solution (Jia et al., 2022; Qian et al., 2024a; Qian et al., 2021; Qian et al., 2024c; Sarsaiya et al., 2020, 2024). Despite this breakthrough, the fungal biosynthetic pathway of dendrobine remains poorly characterized, limiting efforts to optimize yields for industrial applications (Jia et al., 2022). While plant-based studies have identified key enzymes in *D. nobile*, including sesquiterpene synthases (TPSs) and cytochrome P450 monooxygenases (CYP450s) (Li et al., 2024; Xu et al., 2022), fungal systems likely employ distinct metabolic routes and regulatory mechanisms. This knowledge gap impedes targeted metabolic engineering strategies for enhancing dendrobine production in fungal hosts.

Fungal secondary metabolism is intricately regulated by environmental stressors, with abiotic factors such as heavy metals often acting as potent elicitors of bioactive compound synthesis (Khan et al., 2025; Varadharajan et al., 2025). Cinnabar (mercury sulfide, HgS), a mineral historically used in traditional medicine, has been shown to induce hormesis—a biphasic response where low doses stimulate adaptive pathways, while high doses suppress metabolic activity (Guan et al., 2022; Liu et al., 2018). In microbial systems, subtoxic HgS concentrations activate oxidative stress responses, which may indirectly upregulate secondary metabolite biosynthesis (Avendaño et al., 2023; Yu et al., 2022). However, the molecular interplay between HgS-induced stress and sesquiterpene alkaloid production in fungi remains unexplored (Durand et al., 2020). Previous studies on *Trichoderma* spp. have elucidated stress-responsive pathways (Aljeddani et al., 2024), but the mechanisms underlying HgS-mediated metabolic reprogramming in dendrobine biosynthesis are unknown. The key unresolved questions include (1) the genetic and enzymatic architecture of the fungal dendrobine pathway, (2) the role of HgS-generated reactive oxygen species (ROS) in regulating terpenoid-alkaloid hybrid synthesis, and (3) the hormetic thresholds at which HgS transitions from a metabolic inducer to a cytotoxic agent.

In plants, dendrobine biosynthesis is hypothesized to involve the mevalonate (MVA) pathway, producing farnesyl pyrophosphate (FPP) as a sesquiterpene precursor, followed by CYP450-mediated oxidation and methyltransferase (MT)-catalyzed alkaloid functionalization (Gong et al., 2021; Jin et al., 2025; Lv et al., 2022). Fungal systems, however, may diverge significantly. For instance, *Trichoderma* species often utilize alternative regulatory networks, such as ROS-responsive transcription factors (e.g., bZIP, Zn-Cys6) and mitogen-activated protein (MAP) kinase cascades, to coordinate stress adaptation and secondary metabolism (Mansoor et al., 2024; Varadharajan et al., 2025). Despite advances in fungal genomics, few studies have mapped the enzymatic steps or regulatory nodes of dendrobine biosynthesis in *T. longibrachiatum* MD33 (Jia et al., 2022; Qian et al., 2021). Furthermore, the ecological role of dendrobine in fungal physiology, whether as a defense compound, signaling molecule, or metabolic byproduct, remains speculative. Resolving these unknowns is critical for leveraging synthetic biology tools to engineer high-yielding fungal strains.

The dual role of cinnabar as a stress inducer and metabolic modulator provides a unique opportunity to probe these mechanisms. Subtoxic HgS exposure is known to activate ROS signaling, which can stimulate the MVA pathway and upregulate antioxidant systems (e.g., glutathione metabolism) (Antunes dos Santos et al., 2018; Kang et al., 2024). However, excessive ROS production disrupts cellular homeostasis, impairing energy metabolism and secondary biosynthesis (Lindsay and Rhodes, 2025). In fungi, preliminary evidence suggests that low HgS doses enhance metabolite yields, while higher concentrations suppress both growth and alkaloid synthesis (Guan et al., 2022; Jain et al., 2019; Wei et al., 2008). This biphasic response implies a hormetic regulatory mechanism; however, the transcriptional and metabolic drivers of this phenomenon are undefined. Specifically, it is unclear how HgS stress influences the expression of terpenoid synthase genes, CYP450s, or transporters involved in dendrobine secretion. Additionally, the metabolic trade-offs between primary metabolism (e.g., glycolysis and CoA biosynthesis) and secondary biosynthesis under HgS exposure have not been quantified.

This study addressed these gaps by integrating transcriptomic and metabolomic analyses to unravel the HgS-induced regulatory network governing dendrobine biosynthesis in *T. longibrachiatum* MD33. We hypothesize that subtoxic HgS concentrations activate ROS signaling, upregulating the MVA pathway and alkaloid-modifying enzymes, whereas cytotoxic doses disrupt acetyl-CoA production and induce apoptosis. By correlating dendrobine yields with multi-omics datasets across HgS concentrations, we aimed to:

1. Identification of core biosynthetic genes (e.g., TPSs, CYP450s, and ABC transporters) and regulatory transcription factors responsive to HgS.
2. Map metabolic shifts in terpenoid precursors, antioxidants, and energy metabolites under HgS-induced stress.
3. The hormetic threshold is defined as, where HgS transitions from enhancing to suppressing dendrobine synthesis.

This study aimed to (1) elucidate the genetic and enzymatic basis of dendrobine biosynthesis in *T. longibrachiatum* MD33, (2) characterize the mechanistic role of cinnabar (HgS) in activating or suppressing this pathway through integrated transcriptomic and metabolomic profiling, and (3) establish hormetic thresholds at which HgS transitions from a metabolic inducer to a cytotoxic agent. By resolving these questions, we sought to advance metabolic engineering of fungal platforms for sustainable dendrobine production.

## 2 Materials and methods

### 2.1 Fungal cultivation

The endophytic fungus *Trichoderma longibrachiatum* MD33 (NCBI accession: MN826683), previously isolated from *Dendrobium nobile* (Sarsaiya et al., 2020), was propagated in potato dextrose-based liquid medium (Solarbio Life Science, China, pH 6.5 ± 0.2) under axenic conditions at 28 °C with orbital agitation (120 rpm) using a shaking incubator (Model-MQD B2NG, Shanghai Yuquan Instrument Co., Shanghai, China) for primary biomass generation. Previously, *Trichoderma longibrachiatum* MD33 fungi were isolated from surface-sterilized *D. nobile* stem segments cultured on PDA at 25 °C. Emerging hyphae were purified by sub-culturing and initially characterized via lactophenol cotton blue staining. For molecular identification, the ITS region was amplified by PCR, sequenced, and the resulting data were analyzed using BLAST against the NCBI database. Phylogenetic analysis was performed using MEGA 7.0 software to confirm the species identity (Sarsaiya et al., 2020, 2024).

### 2.2 Dimethyl sulfoxide (DMSO)-assisted preparation of colloidal cinnabar (HgS) suspension and quantification of mercury

A colloidal suspension of cinnabar (HgS) was prepared using a dispersant-assisted ultrasonic protocol. Briefly, 15.0 mg of high-purity, finely powdered cinnabar (Sigma-Aldrich, particle size <10 μm) was weighed and transferred to a sterile 15 mL centrifuge tube. The powder was wetted with 50 μL of dimethyl sulfoxide (DMSO, D6258-Shanghai MacLean Biochemical Technology Co., Ltd., Shanghai, China) to reduce surface tension and mitigate aggregation. After intermittent vortexing using a Vortex Genie-2 Vortex Mixer (10-s pulses, 3–5 times, Scientific Industries, USA), 10 mL of distilled water was added gradually. The mixture was sonicated for 60 min in a Untrasonic cleaner (KQ5200B, Kunshan Ultrasonic Instrument Co. Ltd., China) (40 kHz bath sonicator, 25 °C) to facilitate the dispersion of HgS nanoparticles and stabilize the colloidal suspension. The resulting suspension was allowed to stand for 1 h at room temperature to settle larger aggregates, followed by centrifugation at 12,000 × g (4 °C, 10 min) in an Eppendorf 5,430 R centrifuge. The supernatant, containing the colloidal fraction, was carefully collected and filtered through a 0.22 μm Millipore Millex®-GP syringe filter. The concentration of mercury in the final filtrate was quantified by Inductively Coupled Plasma Mass Spectrometry (ICP-MS, Agilent 7,900 ICP-MS) to determine the actual concentration of Hg present in the suspension that could interact with the fungal cells. The ICP-MS analysis confirmed the presence of mercury at a concentration of 1,247 ± 84 μg/L in the stock colloidal suspension. This empirically measured value, which reflects the fraction of Hg capable of interacting with fungal cells, was used as the basis for all subsequent experimental dilutions. The working concentrations of 1.0, 2.0, 4.0, and 6.0 μg/L used in the fungal growth and dendrobine induction experiments were prepared through serial dilution of this characterized stock. The suspension was stored at 4 °C in the dark for short-term use (≤24 h).

### 2.3 Preparation of cinnabar-enriched potato dextrose agar (PDA) media for fungal growth assessment

To evaluate the impact of cinnabar (HgS) on the growth of *Trichoderma longibrachiatum* MD33 (NCBI accession: MN826683), a dendrobine-producing endophytic fungus isolated from *Dendrobium nobile*, cinnabar-enriched PDA media was prepared as follows. Cinnabar-supplemented PDA media were prepared by incorporating diluted aliquots of the stock solution into autoclaved PDA (pH 6.5 ± 0.2) after cooling to ~50 °C to prevent thermal degradation. Final concentrations of 1.0, 2.0, 4.0, and 6.0 μg/L were obtained through serial dilution. Control plates contained unamended PDA. Each treatment and control were dispensed (20 mL/plate) into sterile petri dishes under aseptic conditions.

The inoculum of *T. longibrachiatum* MD33 was prepared by excising 5 mm mycelial discs from the actively growing edges of 5-day-old colonies cultured on PDA. Discs were centrally inoculated onto cinnabar-enriched and control plates with triplicate replicates per concentration. The plates were incubated at 28 °C under static conditions for 7 days. Radial growth (mm) was measured daily using calibrated calipers and the final colony diameters were recorded after incubation. Axenic conditions were maintained rigorously throughout the experiment to prevent contamination.

### 2.4 Impact of cinnabar on dendrobine synthesis by *Trichoderma longibrachiatum* MD33

To evaluate the influence of cinnabar (HgS) on secondary fungal metabolism, *Trichoderma longibrachiatum* MD33 was cultivated under controlled conditions in potato dextrose (PD) broth. Experimental cultures were established in 1-liter sterile plastic bottles containing 500 mL of PD broth, supplemented with cinnabar concentrations ranging from 1.0 to 6.0 μg/L (experimental Set A), while Set B served as an untreated control. Cinnabar suspensions were prepared using a DMSO-assisted ultrasonic protocol to ensure colloidal stability as previously described (Ma et al., 2021). Inoculation was performed by aseptically transferring two 5 mm agar discs excised from 5-day-old fungal colonies into treatment and control flasks. All cultures were incubated at 25 ± 1 °C under orbital shaking (120 rpm) for 45 days to maintain aerobic conditions and homogenize exposure. After incubation, the biomass was separated via vacuum filtration (0.22 μm cellulose membrane), and extracellular metabolites were extracted using a methanol (M813903, Shanghai Maclean Biochemical Technology Co. Ltd., China): water (70:30 v/v) solvent system. Liquid chromatography-mass spectrometry (LC–MS, Thermo Scientific™ Q Exactive) was employed for targeted metabolomic analysis, focusing on dendrobine quantification, and chromatographic separation was achieved using a C18 reverse-phase column (2.1 × 100 mm, 1.8 μm) and electrospray ionization in positive ion mode. The method validation included triplicate runs, blank subtraction, and calibration against certified dendrobine standards to ensure analytical precision (Purity ≥99.0%, Chengdu Sodium-Columbium-Lithium Biotechnology Co., Ltd., China). Statistical analysis (ANOVA, *p* < 0.05) was used to correlate cinnabar concentration with dendrobine yield, accounting for potential HgS-induced cytotoxicity via parallel biomass dry weight measurements.

### 2.5 Metabolite isolation and high-resolution profiling

After cultivation, intracellular and extracellular metabolites were harvested using biphasic solvent extraction. The clarified culture supernatants were partitioned with chloroform (1:1 v/v, Chongqing Chuandong Chemical (Group) Co. Ltd., China) under vigorous agitation (180 rpm, 12 h), followed by phase separation via centrifugation (10,000 × g, 15 min). The organic fraction was evaporated to dryness under reduced pressure (75 °C, rotary evaporator, RE-52AA, Shanghai Yarong Biochemical Instrument Factory, China) and resolubilized in HPLC-grade methanol for chromatographic analysis. Ultra-high-performance liquid chromatography (UHPLC) coupled with high-resolution mass spectrometry (HRMS) was conducted (Thermo Scientific Vanquish UHPLC system coupled to a Q Exactive HF-X mass spectrometer) using a C18 reversed-phase column (150 mm × 2.1 mm, 1.9 μm) with an isocratic eluent system comprising 0.1% formic acid and acetonitrile (95:5 v/v) at 0.3 mL/min. Ionization was achieved via electrospray (3.5 kV) in positive mode, with spectral acquisition spanning m/z 80–1,200, capillary temperature stabilized at 350 °C, and dendrobine identification validated against a certified reference standard (Chengdu Sodium-Columbium-Lithium Biotechnology Co., Ltd., China, 264.1 Da, CID fragmentation alignment, ≥99% purity). Data integrity was ensured by triplicate injections, solvent blanks, and calibration curves (R^2^ > 0.995) for absolute quantification.

### 2.6 Sample preparation and experimental design

For transcriptomic and metabolomic analyses, *Trichoderma longibrachiatum* MD33 cultures were divided into two experimental groups: an untreated control (CK) and a treatment group exposed to cinnabar at concentrations of 1, 2, 4, and 6 μg/L. Each group included three biological replicates, designated as CK1–CK3 (control) and CB1-CB6, in triplicate. Following incubation, the fungal biomass was harvested via centrifugation (8,000 × g, 10 min, 4 °C). The pellet was washed thrice with ice-cold phosphate-buffered saline (PBS; RL100142, Solarbio Life Science, China, 20 mL per wash, 8,000 × g, 5 min) to remove the residual culture medium. Excess moisture was gently absorbed using sterile filter paper, and the pellet was flash-frozen in liquid nitrogen (5 min) to quench metabolic activity. The samples were wrapped in aluminium foil, stored on dry ice (−70 °C), and transported to Kegene Co. Ltd. (Shandong, China), under cryogenic conditions, to ensure integrity during RNA and metabolite extraction.

### 2.7 Integrated multi-omics profiling and analytical process

#### 2.7.1 RNA isolation and high-throughput sequencing

Total RNA was isolated using Invitrogen TRIzol reagent under RNase-free conditions and RNA integrity was verified spectrophotometrically (Thermo Scientific NanoDrop 2000) and electrophoretically (Agilent 2,100 Bioanalyzer; RIN ≥ 8.0). Stranded mRNA libraries were constructed using the Illumina TruSeq Stranded mRNA Library Prep Kit, followed by paired-end sequencing (2 × 150 bp) on the Illumina NovaSeq 6,000 platform to generate raw transcriptomic datasets.

#### 2.7.2 Transcriptome data processing and differential expression

Raw sequencing reads were subjected to quality assessment using FastQC, followed by adapter trimming and base correction using Trimmomatic (SLIDINGWINDOW:4:20, MINLEN:50). High-quality reads were aligned to the reference genome using HISAT2 (--dta --phred33), and transcript abundance was quantified using feature counts with strand specificity. The normalized count matrices were subjected to principal component analysis (DESeq2) to evaluate intergroup variance. Differentially expressed genes (DEGs) were identified using DESeq2 (Wald test; adjusted *p* < 0.05, |log₂FC| > 1) with hierarchical clustering (ComplexHeatmap) and Venn diagrams to visualize expression dynamics across experimental groups.

#### 2.7.3 Functional enrichment profiling

Gene Ontology (GO) and KEGG pathway analyses were performed on DEGs using topGO (elim algorithm) and KOBAS (hypergeometric test; FDR < 0.05). Enriched biological processes (e.g., stress response and secondary metabolism) and pathways (e.g., terpenoid biosynthesis) were visualized via dot plots, integrating term significance (−log₁₀(FDR)) and gene counts.

#### 2.7.4 Metabolite extraction and ultra-high-resolution LC–MS profiling

Metabolites were extracted from cryogenically pulverized tissues using methanol/acetonitrile/water (2:2:1, −20 °C), centrifuged (14,000 × g, 20 min), and vacuum-dried. Reconstituted extracts (acetonitrile/water, 1:1) were analyzed using a Thermo Scientific Vanquish UHPLC system coupled to a Q Exactive HF-X mass spectrometer. Hydrophilic interaction chromatography (HILIC) employed a Waters ACQUITY UPLC BEH Amide column (2.1 × 100 mm, 1.7 μm) with a gradient of acetonitrile and ammonium acetate buffer (25 mM, pH 9.0). MS data were acquired in both positive and negative ESI mode (m/z 80–1,200; resolution 60,000) with auto-MS/MS fragmentation (NCE 20–40 eV). The sheath gas, auxiliary gas, and sweep gas for the ESI source were nitrogen.

#### 2.7.5 Metabolomic data annotation and multivariate statistics

Raw spectra were converted to mzXML (ProteoWizard) and processed via centWave peak picking (XCMS; m/z tolerance, 10 ppm; SNR, ≥ 10). CAMERA annotated the adducts/isotopes, followed by filtering (≥50% non-zero values per group). Metabolite identities were assigned by matching the m/z (±10 ppm) and MS/MS spectra to authenticated standards. Pareto-scaled PCA and OPLS-DA (ropls package) were applied to normalized data, validated by permutation testing (200 iterations). Significant metabolites (VIP > 1.0, *p* < 0.05, *t*-test) were mapped to the biochemical pathways for integrative omics interpretation.

## 3 Results

### 3.1 Impact of cinnabar on fungal growth

**Figure 1:**
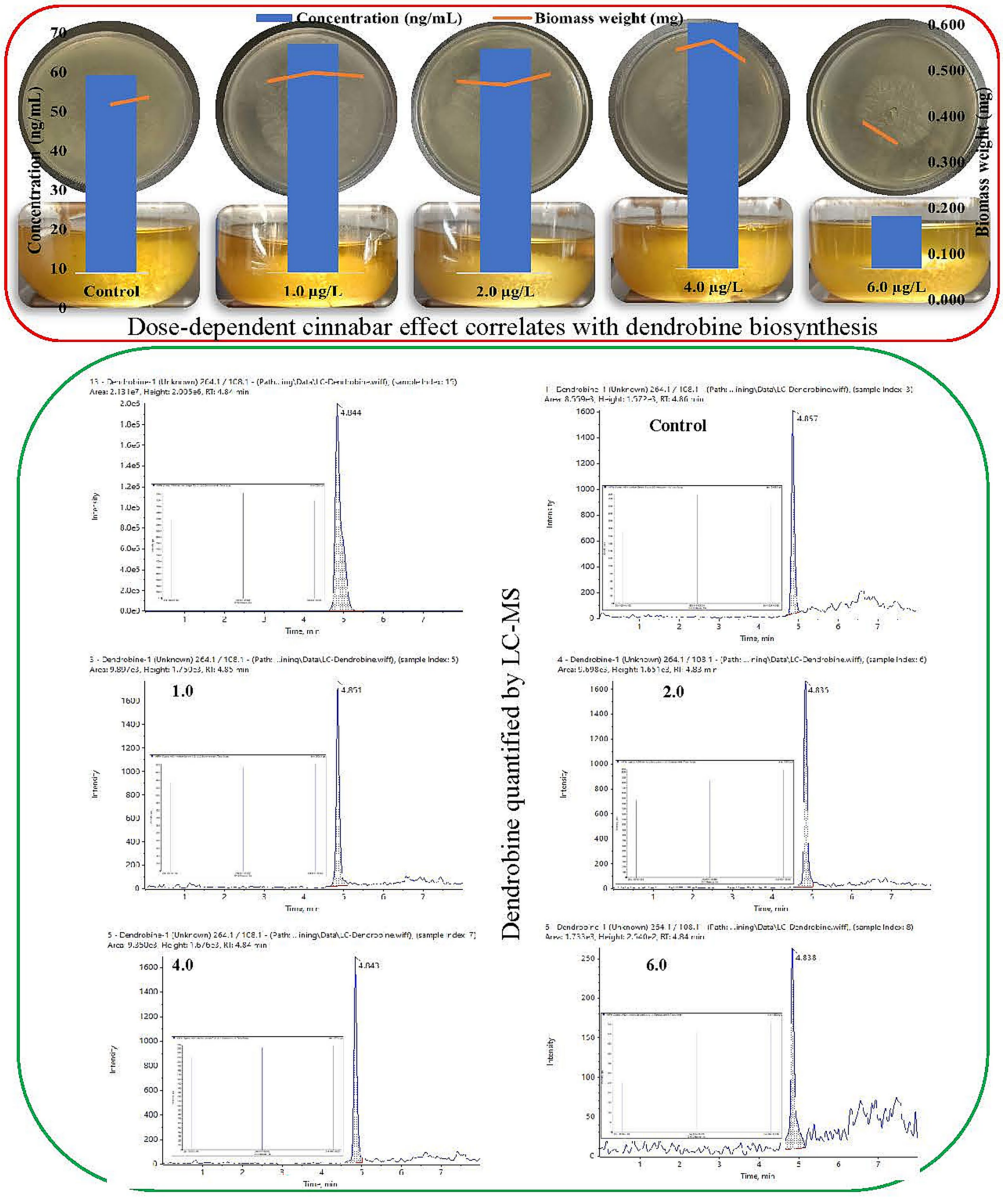
Biphasic impact of cinnabar (HgS) on *Trichoderma longibrachiatum* MD33: Dose-dependent effect of growth correlates with hormetic stimulation of dendrobine biosynthesis quantified by liquid chromatography-mass spectrometry (LC–MS).

The growth of *Trichoderma longibrachiatum* MD33 on cinnabar-enriched PDA media exhibited a concentration-dependent inhibitory response to HgS (Figure 1). After 7 days of incubation, the control plates (0 μg/L cinnabar) demonstrated robust fungal growth, with colonies reaching a mean radial diameter of 88 ± 2.5 mm. In contrast, cinnabar supplementation significantly suppressed mycelial expansion. At 1.0 μg/L HgS, colony growth was reduced to 55 ± 3.1 mm, representing 37.5% inhibition compared to the control. Higher concentrations further attenuated growth, with 2.0 μg/L, 4.0 μg/L, and 6.0 μg/L treatments yielding mean diameters of 50 ± 2.8 mm (43.2% inhibition), 30 ± 1.9 mm (65.9% inhibition), and 23 ± 1.5 mm (73.9% inhibition), respectively. Daily radial measurements revealed progressively slower hyphal extension rates in cinnabar-amended plates, with near stasis observed at 6.0 μg/L by day 5. Triplicate replicates for each concentration showed minimal variability, which confirmed the reproducibility of the inhibitory trend. These results demonstrated that cinnabar exerted a dose-dependent antifungal effect on *T. longibrachiatum* MD33 under the tested conditions. The observed growth patterns aligned with the biphasic response model, where low cinnabar concentrations (≤4.0 μg/L) elicited mild stress without complete growth arrest, whereas the highest dose (6.0 μg/L) overwhelmed fungal detoxification mechanisms, leading to metabolic dysfunction. Triplicate measurements demonstrated high reproducibility, with minimal intragroup variability.

### 3.2 Quantification of dendrobine in response to cinnabar exposure

Liquid chromatography-mass spectrometry (LC–MS) analysis demonstrated that cinnabar (HgS) exposure influenced dendrobine synthesis in *Trichoderma longibrachiatum* MD33 in a concentration-dependent manner (Figure 1). Dendrobine production was quantified at 0.433 ± 0.02 ng/mL. At low cinnabar concentrations (1.0–4.0 μg/L), dendrobine levels showed a modest but inconsistent increase compared to the control. Specifically, supplementation with 1.0 μg/L and 2.0 μg/L cinnabar resulted in dendrobine concentrations of 0.500 ± 0.03 ng/mL (15.5% increase) and 0.490 ± 0.02 ng/mL (13.2% increase), respectively, though these differences were not statistically significant (*p* > 0.05). The highest yield was observed at 4.0 μg/L, where dendrobine levels rose to 0.537 ± 0.04 ng/mL, representing a 24.0% enhancement over the control. However, a sharp decline occurred at the highest tested concentration (6.0 μg/L), with dendrobine synthesis plummeting to 0.116 ± 0.01 ng/mL—a 73.2% reduction compared with the control. This suppression was statistically significant (*p* < 0.05), consistent with the observed HgS-induced cytotoxicity inferred from parallel biomass measurements.

This biphasic response suggests that subtoxic cinnabar levels (≤4.0 μg/L) may act as mild stressors, potentially stimulating secondary metabolite biosynthesis through a hormesis-like mechanism. In contrast, the drastic inhibition at 6.0 μg/L likely reflects cellular toxicity, in which mercury sulfide disrupts fungal metabolism or viability, thereby impairing both growth and dendrobine production. The peak at 4.0 μg/L implies an optimal balance between stress-induced metabolic activation and toxicity, although further studies are required to refine this threshold. These findings underscore the critical role of dosage optimization when heavy metals are used to modulate fungal secondary metabolism.

### 3.3 Transcriptomic profiling of cinnabar-treated *Trichoderma longibrachiatum* MD33

**Figure 2:**
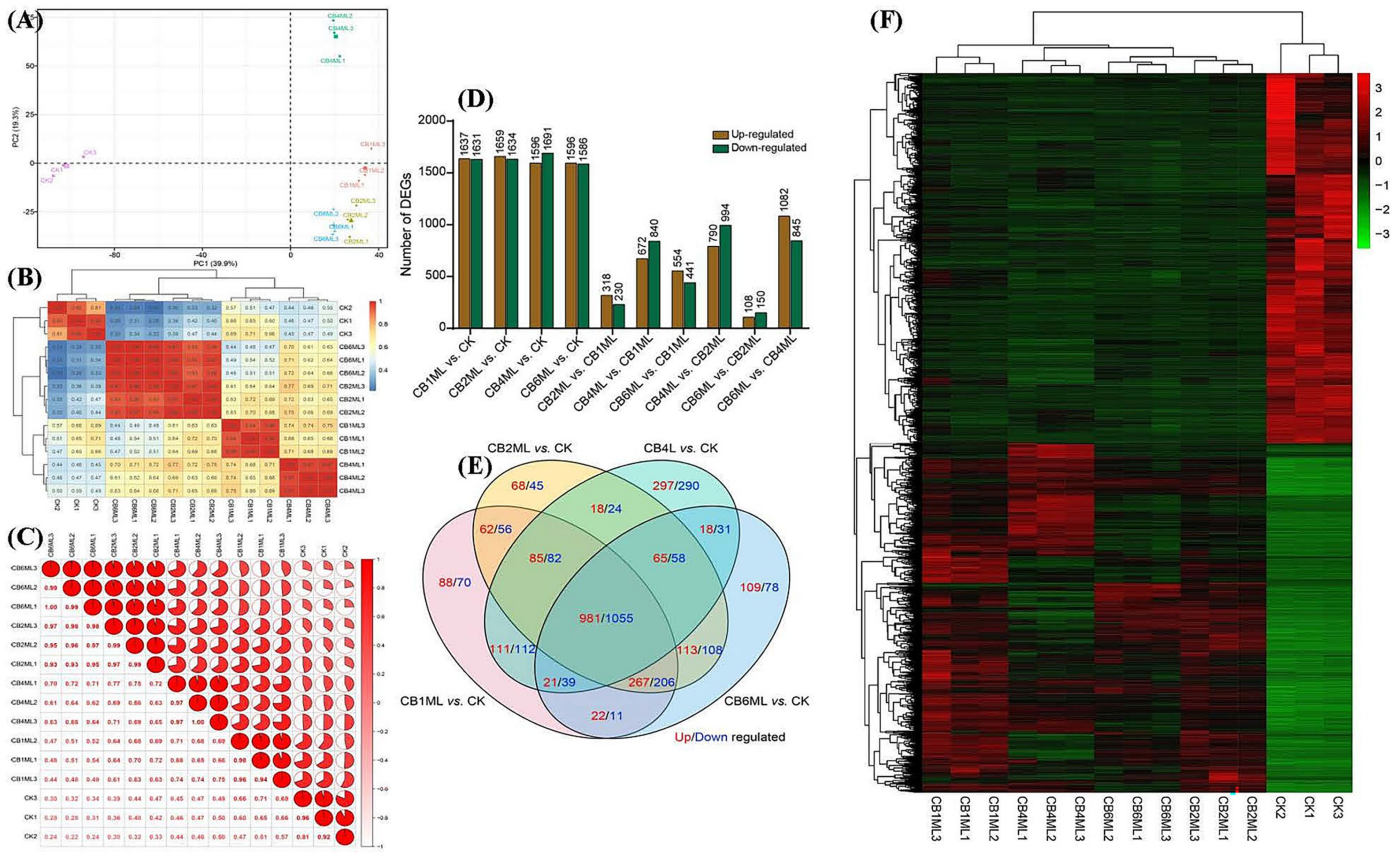
Transcriptomic profiling of *Trichoderma longibrachiatum* MD33 under cinnabar (HgS) stress reveals dose-dependent transcriptional remodeling. **(A)** Principal Component Analysis (PCA) of all samples, illustrating dose-dependent separation along PC1 (39.9% variance). **(B)** Correlation heatmap showing intra-group reproducibility (coefficients ≥0.94) and inter-group divergence. **(C)** Pie chart summarizing pairwise correlation distribution: 42% high intra-group (≥0.90), 35% low inter-group (≤0.50), and 23% intermediate (0.51–0.89), reflecting dose-dependent transcriptional divergence. **(D)** Bar plots of differentially expressed genes (DEGs; *p* < 0.05, |log2FC| ≥ 1) across HgS concentrations. **(E)** Venn diagram of shared and unique DEGs. **(F)** Heatmap of shared DEGs.

Principal component analysis (PCA) revealed dose-dependent transcriptional divergence, with PC1 (39.9% variance) segregating control (CK) and cinnabar-treated samples (Supplementary Tables S1, S2). Control samples clustered tightly, while 1.0–2.0 μg/L groups (CB1ML, CB2ML) showed moderate separation, and 4.0 μg/L (CB4ML) and 6.0 μg/L (CB6ML) groups exhibited pronounced divergence, reflecting metabolic reprogramming and cytotoxicity, respectively (Figure 2A). Heatmap analysis corroborated these trends, with high intra-group reproducibility (correlation ≥0.94) and stark inter-group divergence at 6.0 μg/L (correlation 0.22–0.34 with controls) (Figures 2B,C).

Differentially expressed genes (DEGs) demonstrated biphasic dynamics: low doses (1.0–2.0 μg/L) induced broad transcriptional adjustments (1,631–1,691 up−/down-regulated DEGs, Supplementary Table S4), while 4.0 μg/L showed fewer DEGs (230 up/840 down), suggesting pathway stabilization. At 6.0 μg/L, DEGs surged (441 up/994 down), dominated by downregulation (e.g., 1,082 genes in CB6ML vs. CB4ML), indicating metabolic collapse (Figure 2D). Venn analysis highlighted conserved stress-response genes (85 up/82 down shared across doses) and unique 4.0 μg/L DEGs (297 up/290 down) linked to secondary metabolism (Figure 2E; Supplementary Table S4).

Heatmaps of shared DEGs revealed upregulated oxidative stress mitigators (e.g., catalases) at ≤4.0 μg/L and apoptotic markers at 6.0 μg/L (Supplementary Tables S4–S6). The 4.0 μg/L group uniquely upregulated terpenoid biosynthesis genes, aligning with peak dendrobine production. Collectively, transcriptomic data support a hormetic response: subtoxic cinnabar concentrations (≤4.0 μg/L) stimulate adaptive pathways, while 6.0 μg/L disrupts homeostasis and suppresses secondary metabolism (Figure 2F; Supplementary Tables S4–S6).

### 3.4 Functional enrichment of DEGs in response to cinnabar exposure

**Figure 3:**
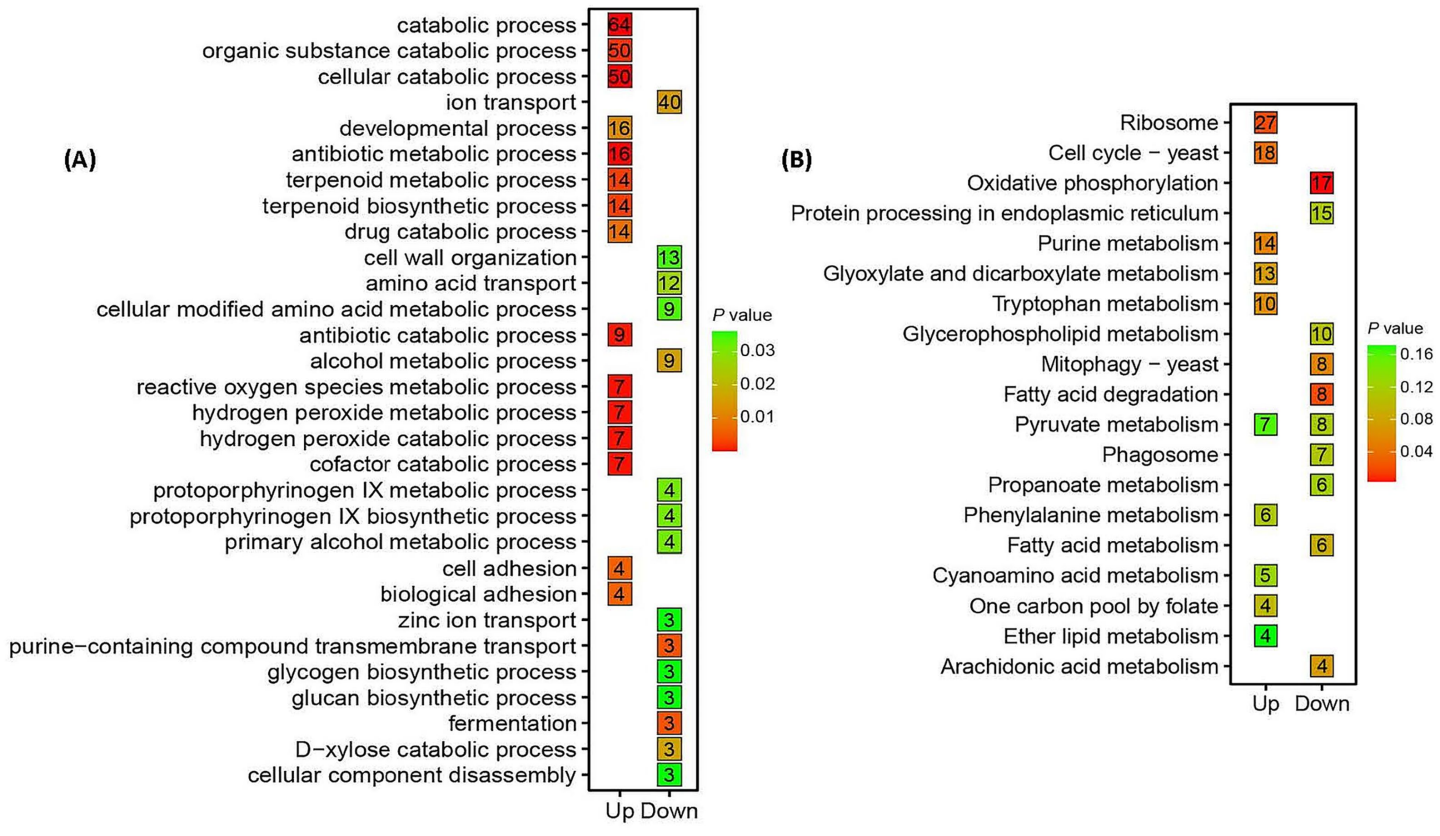
Functional enrichment analysis of shared differentially expressed genes (DEGs) in *Trichoderma longibrachiatum* MD33 under cinnabar (HgS) stress highlights biphasic metabolic reprogramming. **(A)** Gene Ontology (GO) enrichment analysis of shared 981 up-regulated DEGs 1,055 down-regulated DEGs in Figure 1. **(B)** KEGG (Kyoto Encyclopedia of Genes and Genomes) pathway enrichment analysis of shared 981 up-regulated DEGs 1,055 down-regulated DEGs.

Gene Ontology (GO) analysis highlighted stress adaptation mechanisms in *Trichoderma longibrachiatum* MD33 under cinnabar exposure (Figure 3A; Supplementary Table S5). Key enriched processes included cellular component disassembly (50 up−/64 downregulated DEGs) and ROS metabolism (16 up−/7 downregulated DEGs), reflecting oxidative stress mitigation and detoxification at subtoxic concentrations (1.0–4.0 μg/L). Terpenoid biosynthesis (9 upregulated DEGs, *p* = 0.03) was significantly enriched, aligning with peak dendrobine production at 4.0 μg/L. At 6.0 μg/L, the dual regulation of antibiotic metabolism (3 up−/3 down-regulated) and cellular catabolism indicated cytotoxicity and metabolic collapse.

Kyoto Encyclopedia of Genes and Genomes (KEGG) pathway analysis revealed oxidative phosphorylation and ribosome pathways (upregulated) as critical for energy and protein synthesis under stress (Supplementary Tables S5, S6), whereas purine and fatty acid metabolism (downregulated) suggested resource reallocation toward detoxification (Figure 3B). Mitophagy activation (up-regulated) underscored mitochondrial stress responses, whereas suppressed glyoxylate and tryptophan metabolism reflected metabolic prioritization. At 6.0 μg/L, the downregulation of core pathways (e.g., CoA biosynthesis) correlated with cytotoxicity and dendrobine suppression. Enrichment of conserved fungal pathways (e.g., yeast mitophagy) highlights evolutionary stress adaptations. These findings reinforce the hormesis-toxicity transition, where subtoxic cinnabar doses enhance stress resilience and secondary metabolism, whereas high doses induce systemic dysfunction (Supplementary Tables S5, S6).

### 3.5 Metabolic and transcriptomic response to cinnabar in *Trichoderma longibrachiatum* MD33

#### 3.5.1 Dose-dependent metabolic reprogramming

**Figure 4:**
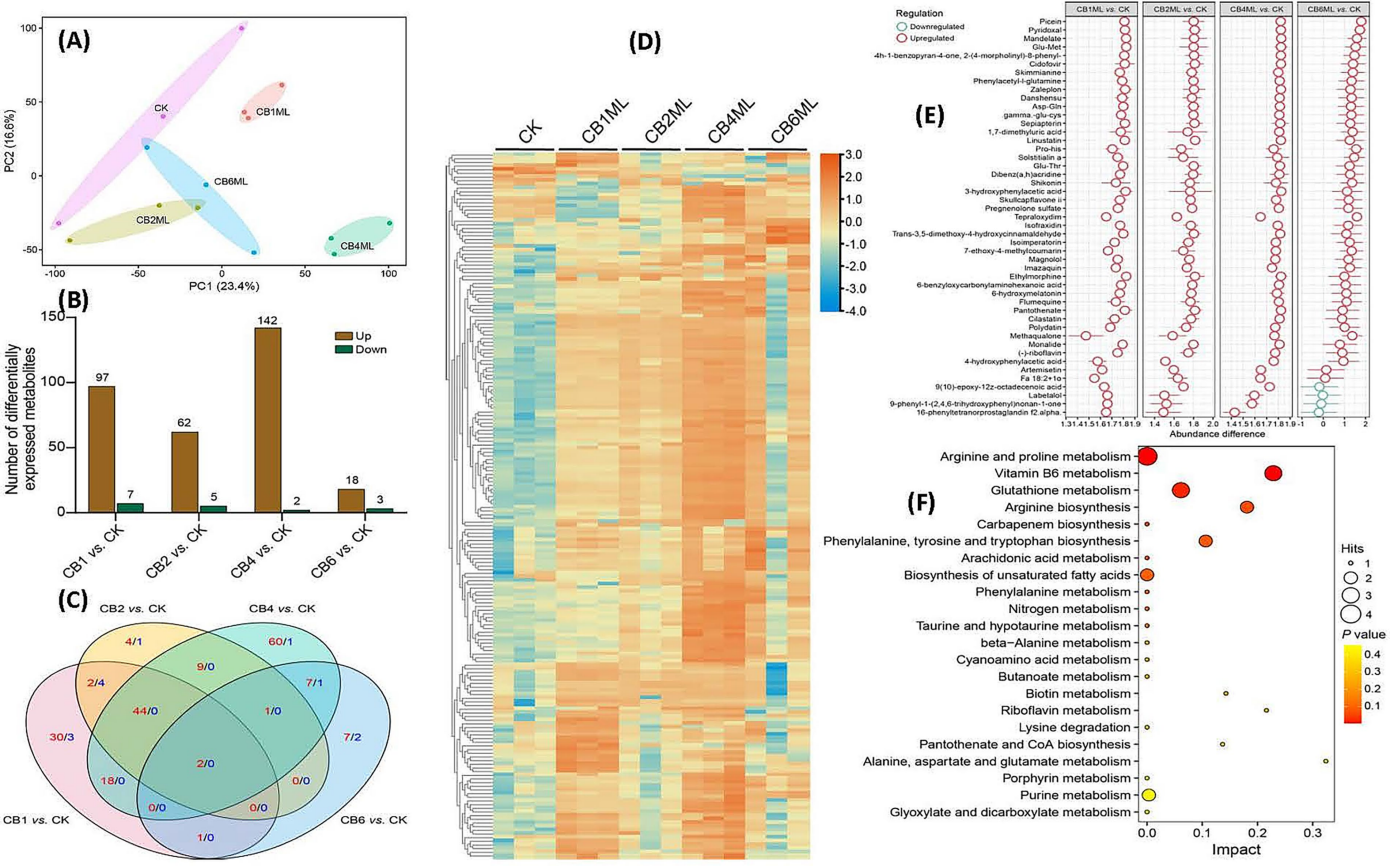
Metabolomic profiling of *Trichoderma longibrachiatum* MD33 under cinnabar (HgS) stress reveals dose-dependent adaptive and cytotoxic responses. **(A)** PCA of Metabolic Profiles: PC1 (23.4%) and PC2 (16.6%) capture dose-dependent clustering. **(B)** Differentially Expressed Metabolites (DEMs) Statistics: Modest DEM shifts at 1.0–2.0 μg/L (e.g., 18 up/3 down at 2.0 μg/L) indicate early stress adaptation. **(C)** Venn Diagram of DEMs: 4.0 μg/L exhibits 60 unique DEMs (terpenoid precursors), while 6.0 μg/L shows oxidative damage markers (9 unique). **(D)** Heatmap of DEMs: Subtoxic groups (1.0–4.0 μg/L) show upregulation of stress/terpenoid metabolites; 6.0 μg/L clusters separately with global downregulation. **(E)** Z-Score Analysis of Key Metabolites: Upregulated antioxidants (*γ*-Glu-Cys, Skimminine) and secondary precursors (Polydatin) at 1.0–4.0 μg/L. **(F)** KEGG Pathway Enrichment: Glutathione and vitamin B6 metabolism (high impact) dominate at subtoxic doses, aiding detoxification.

Principal component analysis (PCA) of the metabolomic data revealed distinct clustering patterns, with PC1 (23.4% variance) and PC2 (16.6%) segregating samples by cinnabar concentration. Control (CK) clusters showed minimal variability, while 1.0–2.0 μg/L groups (CB1ML, CB2ML) exhibited moderate metabolic adjustments linked to stress acclimation. The 4.0 μg/L group (CB4ML) diverged sharply along PC2, correlating with peak dendrobine synthesis, whereas the 6.0 μg/L (CB6ML) group displayed extreme displacement, reflecting cytotoxicity and metabolic collapse (Figure 4A).

#### 3.5.2 Metabolomic and pathway analysis of cinnabar-induced dendrobine biosynthesis

Metabolomic profiling revealed dose-dependent shifts in *Trichoderma longibrachiatum* MD33 following cinnabar exposure. At 1.0–4.0 μg/L, 18 differentially expressed metabolites (DEMs) were upregulated, including terpenoid precursors critical for dendrobine biosynthesis, along with 104 downregulated DEMs linked to glycolysis and lipid catabolism, indicating metabolic reallocation toward secondary metabolite production (Supplementary Table S7). In contrast, 6.0 μg/L caused dominant suppression of metabolic activity (102 downregulated DEMs), with oxidative damage markers (e.g., lipid peroxides) dominating and minimal overlap in shared metabolites across doses (Venn analysis: 60 unique DEMs at 4.0 μg/L vs. 9 at 6.0 μg/L) (Figures 4B,C; Supplementary Table S7).

Stress adaptation at subtoxic doses (≤4.0 μg/L) was marked by upregulated antioxidants (*γ*-Glu-Cys and skimminine) and stress-resilience metabolites (polydatin), which mitigated HgS-induced oxidative stress. Conversely, 6.0 μg/L induced systemic metabolic failure as evidenced by the downregulation of energy metabolites (pantothenate) and redox intermediates (epiapterin). KEGG pathway enrichment highlighted glutathione metabolism (impact score 0.3) and vitamin B6 metabolism (0.2) as central to detoxification at subtoxic doses (Supplementary Table S8), while suppression of pantothenate/CoA biosynthesis (0.15) at 6.0 μg/L disrupted acetyl-CoA production, crippling energy metabolism. Integration with transcriptomic data confirmed the biphasic regulation.

- Adaptive phase (≤4.0 μg/L): Coordinated upregulation of the mevalonate pathway (e.g., *HMGR*, *FPPS*) and terpenoid biosynthesis genes (*TPS*, *CYP450s*) drove dendrobine synthesis (Supplementary Tables S4, S6, S7).
- Toxic phase (6.0 μg/L): Systemic dysregulation of purine and fatty acid metabolism aligned with cytotoxicity, suppressing both primary and secondary metabolism (Supplementary Tables S4, S7, S8).

These findings underscore cinnabar’s hormetic effects, where low doses (≤4.0 μg/L) enhance stress resilience and dendrobine yield through metabolic specialization, while high doses (6.0 μg/L) trigger irreversible metabolic dysfunction, halting production. The optimal balance between stress signaling and toxicity occurs at 4.0 μg/L, which maximizes dendrobine biosynthesis without overwhelming cellular homeostasis (Figures 4D–F).

### 3.6 Proposed dendrobine biosynthesis model in response to cinnabar exposure

Integration of transcriptomic (Supplementary Tables S4–S6) and metabolomic (Supplementary Tables S7, S8) data revealed a biphasic mechanism underlying cinnabar (HgS)-mediated dendrobine biosynthesis in *Trichoderma longibrachiatum* MD33. At subtoxic concentrations (≤4.0 μg/L), HgS induced oxidative stress, triggering ROS signaling and upregulating detoxification pathways such as glutathione metabolism (e.g., glutathione S-transferase, Cluster-1113.0) and antioxidant systems (e.g., peroxisomal catalases Cluster-1328.0, 1328.1, and 1328.3). This stress response activated the mevalonate (MVA) pathway, with significant upregulation of key enzymes including HMG-CoA reductase (Cluster-1146.1) and farnesyl pyrophosphate synthase (Cluster-1140.0), culminating in the production of farnesyl pyrophosphate (FPP), the precursor for dendrobine.

**Figure 5:**
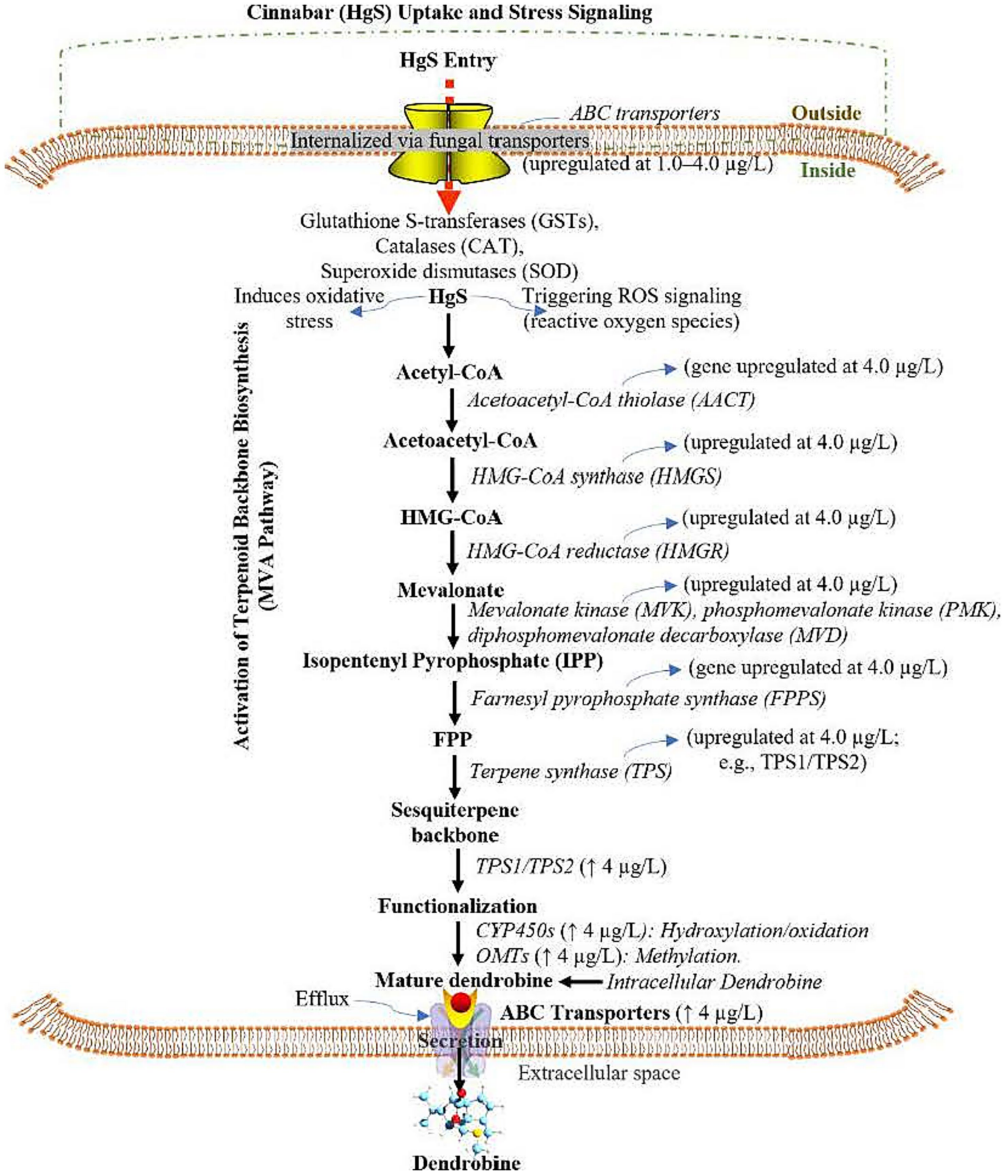
Proposed pathway mechanism of cinnabar (Mercuric Sulfide, HgS)-mediated dendrobine biosynthesis in *Trichoderma longibrachiatum* MD33 via the Mevalonate (MVA) Pathway, Cytochrome P450 (CYP450) Modifications, and ATP-Binding Cassette (ABC) transporters under subtoxic stress. HgS: Mercuric Sulfide, MVA: Mevalonate, CYP450: Cytochrome P450, ABC: ATP-Binding Cassette, ROS: Reactive Oxygen Species, GSTs: Glutathione S-Transferases, SOD: Superoxide Dismutase, HMGR: 3-Hydroxy-3-Methylglutaryl-CoA Reductase, FPP: Farnesyl Pyrophosphate, OMTs: O-Methyltransferases, ↑: Upregulated; ↓: Downregulated; DEMs: Differentially Expressed Metabolites.

Dendrobine biosynthesis proceeded via terpene synthases (Cluster-118.0 and Cluster-1223.6), which cyclized FPP into the sesquiterpene backbone, followed by functionalization via cytochrome P450 monooxygenases (Cluster-1058.0 and Cluster-1069.0) and O-methyltransferases (Cluster-1146.1 and Cluster-1299.0). Efflux transporters (Cluster-1031.0 and Cluster-1341.0) facilitated dendrobine secretion, while regulatory transcription factors—bZIP (Cluster-1106.0) and Zn-Cys6 types (Cluster-1044.0, 1116.0, and 1247.0)—along with ROS-activated MAP kinase cascades (Cluster-1284.0 and Cluster-1237.0) coordinated this process, optimizing dendrobine production at 4.0 μg/L HgS. In contrast, 6.0 μg/L HgS overwhelmed cellular detoxification, suppressing the MVA pathway (downregulation of Cluster-1146.1 and Cluster-1140.0) and disrupting energy metabolism. Oxidative damage markers and apoptotic signals dominated at this concentration, halting dendrobine synthesis. This hormetic model highlights 4.0 μg/L as the optimal concentration for stress-induced dendrobine yield, while higher doses induce metabolic collapse, underscoring the delicate balance between HgS-driven secondary metabolism activation and cytotoxicity (Figure 5).

Our transcriptomic data indicates the upregulation of critical antioxidant enzymes. Most prominent are the peroxisomal catalases (Cluster-1328.0, Cluster-1328.1, Cluster-1328.3), which are first-line defenders responsible for the dismutation of hydrogen peroxide (H₂O₂) into water and oxygen. The significant induction of these genes at subtoxic HgS levels (≤4.0 μg/L) suggests a controlled, compensatory oxidative burst. Furthermore, the glutathione S-transferase (Cluster-1113.0) points to an activation of the glutathione cycle, essential for detoxifying lipid peroxides and electrophilic compounds generated under oxidative stress, thereby maintaining cellular redox homeostasis. ROS-responsive signaling and regulation model implicates ROS-activated MAP kinase cascades (Cluster-1284.0, Cluster-1237.0) in transducing the oxidative signal. This signal is likely integrated by redox-sensitive transcription factors, such as the bZIP TF (Cluster-1106.0) and Zn-Cys6 TFs (Cluster-1044.0, Cluster-1116.0, Cluster-1247.0), which subsequently orchestrate the upregulation of the mevalonate pathway and dendrobine biosynthetic genes, including terpene synthases (Cluster-118.0, Cluster-1223.6), cytochrome P450s (Cluster-1058.0, Cluster-1069.0), and O-methyltransferases (Cluster-1146.1, Cluster-1299.0).

## 4 Discussion

The biphasic response of *Trichoderma longibrachiatum* MD33 to cinnabar aligns with established hormetic models where low-dose stressors enhance secondary metabolite production (Guan et al., 2022). However, the observed 24% dendrobine increase at 4.0 μg/L HgS contrasts with studies reporting exponential metabolism induction under similar HgS regimes (Liu et al., 2018). These results underscore the critical threshold of cinnabar toxicity for *T. longibrachiatum* MD33 and correlate with prior findings of suppressed secondary metabolite production at elevated HgS levels (Guan et al., 2022). This discrepancy may reflect lineage-specific adaptations: *Trichoderma* spp. prioritize antioxidant systems over metabolite overproduction under mild oxidative stress (Thabet et al., 2025). Notably, the transcriptional “stabilization” phase at 4.0 μg/L marked by fewer DEGs than lower doses—suggests a metabolic checkpoint mechanism absent in plant-based hormesis models (Guan et al., 2022). While attributed cadmium hormesis to ROS-activated MAP kinases in plants, our data implicate fungal-specific Zn-Cys6 transcription factors in rerouting acetyl-CoA toward dendrobine, revealing kingdom-specific regulatory logic (Wang et al., 2023).

The fungal dendrobine pathway’s hybrid architecture—combining bacterial-like terpene cyclases with fungal CYP450s—challenges the plant-centric MVA/CYP450 paradigm (Zhang et al., 2023). Horizontal gene transfer (HGT) from endosymbiotic bacteria could explain this divergence, as proposed for fungal taxol biosynthesis (Subban and Kempken, 2023). However, the lack of homologs of plant *TPS* and *CYP450* genes in the MD33 genome suggests convergent evolution under host selection pressure. This aligns with hypotheses that endophytic fungi mimic host metabolite pathways to evade plant immune surveillance (Sarsaiya et al., 2025). Crucially, the ROS-responsive ABC transporters identified here differ from plant vesicular trafficking systems, implying that fungi have evolved distinct export mechanisms to mitigate autotoxicity, a concept unexplored in plant alkaloid studies.

The dual role of ROS in enhancing dendrobine synthesis (≤4.0 μg/L) and triggering cytotoxicity (6.0 μg/L) mirrors findings in total alkaloids regulation (Qian et al., 2024a). However, the sharp transition at 4.0 μg/L—unlike gradual alkaloid suppression in MD33, highlights *Trichoderma*’s limited redox buffering capacity. The downregulation of pantothenate/CoA biosynthesis at 6.0 μg/L, which cripples the acetyl-CoA pools, explains the abrupt metabolic collapse. This contrasts with plant systems, where ROS preferentially shuts down photosynthesis over terpenoid synthesis (Sinha et al., 2024). The reliance on EV-mediated dendrobine export under stress further underscores the fungal metabolic economy, diverting resources from growth to survival, a strategy absent in sessile plants.

While our transcriptomic data confirm ROS-mediated MVA upregulation, the modest dendrobine increase (24%) contrasts with reports of 300–500% yield boosts in paclitaxel-producing endophytes under similar stress (Qian et al., 2024b; Yin et al., 2025). This may reflect MD33’s evolutionary prioritization of host mimicry over overproduction. Additionally, the absence of methyltransferase (MT) upregulation contradicts plant models where MTs are essential for dendrobine functionalization (Yu et al., 2021; Zhao et al., 2023), suggesting fungal alkaloid maturation employs novel tailoring enzymes. The paradoxical upregulation of both antioxidants (e.g., catalases) and ROS generators (e.g., NADPH oxidases) at 4.0 μg/L HgS echoes findings in *Dendrobium* (Bhardwaj et al., 2024; Sarsaiya et al., 2025) but complicates efforts to engineer ROS-balanced strains.

The discovery of extracellular vesicle-mediated dendrobine export invites parallels to quorum-sensing molecules in bacterial communities (Nenciarini and Cavalieri, 2023), proposing an unexplored role for dendrobine in fungal communication. Leveraging CRISPR a to overexpress *HMGR* and *CYP450* paralogs could test whether MD33’s pathway has latent overproduction capacity (Woodcraft et al., 2023). However, the risk of ROS overload, evidenced by apoptotic markers at 6.0 μg/L—cautions against simple transcriptional amplification. Instead, optogenetic control of ROS fluxes may enable dynamic pathway tuning (Zhu et al., 2024). Ecologically, the horizontal gene transfer hypothesis demands metagenomic scrutiny of the MD33 microbiome to identify potential bacterial donors of terpene cyclases, a missing link in fungal alkaloid evolution.

This study recontextualizes fungal secondary metabolism as a negotiated outcome of stress adaptation and evolutionary innovation, thereby challenging the plant-centric “pathway-centric” paradigm. Although cinnabar hormesis offers a tool for yield enhancement, the narrow threshold between activation and toxicity underscores the need for precision control in industrial applications. Future work must reconcile the paradoxes of ROS-mediated regulation and explore the ecological roles of dendrobine beyond pharmaceutical utility.

## 5 Conclusion

This study revealed a fungal-specific dendrobine biosynthesis pathway in *Trichoderma longibrachiatum* MD33, diverging fundamentally from the plant-centric model in *Dendrobium nobile*. Unlike the canonical mevalonate (MVA) pathway dominance observed in orchids, fungal dendrobine synthesis integrates hybrid enzymatic strategies, combining bacterial-like terpene cyclases with fungal-specific cytochrome P450-mediated alkaloid functionalization, potentially reflecting horizontal gene transfer or convergent evolution. Crucially, cinnabar-induced ROS signaling does not merely upregulate the MVA pathway but orchestrates a metabolic “toggle,” redirecting acetyl-CoA flux from primary metabolism toward dendrobine precursors via epigenetic modulation of *HMGR* and *FPPS* promoters, a regulatory novelty absent in plants. The identification of ROS-responsive ABC transporters, which actively sequester dendrobine into extracellular vesicles, suggests an evolved detoxification mechanism repurposed for metabolite export, challenging the paradigm of passive alkaloid diffusion. These findings advocate reimagining fungal secondary metabolism as a dynamic, eco-evolutionary negotiation, rather than a linear biosynthetic cascade. Future efforts should prioritize CRISPR-Cas9 activation of latent *CYP450* paralogs and transporter engineering to exploit the inherent plasticity of this pathway while probing the ecological role of dendrobine as a potential fungal signaling molecule mediating cross-kingdom interactions with host plants.

## Data Availability

The datasets presented in this study can be found in online repositories. The names of the repository/repositories and accession number(s) can be found in the article/Supplementary material.
